# Why estimands are needed to define treatment effects in clinical trials

**DOI:** 10.1186/s12916-023-02969-6

**Published:** 2023-07-27

**Authors:** Oliver N. Keene, Helle Lynggaard, Stefan Englert, Vivian Lanius, David Wright

**Affiliations:** 1 KeeneONStatistics, Maidenhead, UK; 2 Biostatistics, Novo Nordisk A/S, Bagsværd, Denmark; 3 Statistical Modeling & Methodology, Janssen R&D, Janssen-Cilag GmbH, Neuss, Germany; 4 Statistics & Data Insights, Bayer AG, Wuppertal, Germany; 5 Statistical Innovation, Data Science & Artificial Intelligence, Biopharmaceuticals R&D, AstraZeneca, Cambridge, UK

**Keywords:** Estimand, Treatment effect, Intercurrent event, ITT, Per-protocol, PICO, CONSORT

## Abstract

**Background:**

The estimand for a clinical trial is a precise definition of the treatment effect to be estimated. Traditionally, estimates of treatment effects are based on either an ITT analysis or a per-protocol analysis. However, there are important clinical questions which are not addressed by either of these analyses. For example, consider a trial where patients take a rescue medication. The ITT analysis includes data after use of rescue, while the per-protocol analysis excludes these patients altogether. Neither of these analyses addresses the important question of what the treatment effect would have been if patients did not take rescue medication.

**Main text:**

Trial estimands provide a broader perspective compared to the limitations of ITT and per-protocol analysis. Trial treatment effects depend on how events occurring after treatment initiation such as use of alternative medication or discontinuation of the intervention are included in the definition. These events can be accounted for in different ways, depending on the clinical question of interest.

**Conclusion:**

The estimand framework is an important step forward in improving the clarity and transparency of clinical trials. The centrality of estimands to clinical trials is currently not reflected in methods recommended by the Cochrane group or the CONSORT statement, the current standard for reporting clinical trials in medical journals. We encourage revisions to these guidelines.

## Background

The CONSORT statement is used worldwide for the reporting of randomised controlled trials [1, 2]. While CONSORT requires specification of the outcome measure, this does not provide a precise definition of the treatment effect. Estimation of the treatment effect is typically complicated by events that occur after initiation of treatment such as use of alternative medications or discontinuation of the assigned treatment. Such events have been termed intercurrent events, defined as “events occurring after treatment initiation that affect either the interpretation or the existence of the measurements associated with the clinical question of interest” [3].

In their recent paper, Little and Lewis [4] use a different definition of a trial estimand, referring to this simply as the “true effect of the intervention”. However, there are alternative ways in which the treatment effect can be defined as it depends on how events such as use of alternative medication or discontinuation of the intervention are included in the definition and therefore there is no single “true” treatment effect.

For example, PIONEER-1 [5] compared the effects of semaglutide with placebo on glycaemic control in patients with type 2 diabetes mellitus. Rescue medication was recommended for persistent and unacceptable hyperglycaemia, and it was expected that more placebo patients would need rescue medication compared to semaglutide. Is the true effect of treatment the effect of semaglutide including the potential effects of differential use of rescue medication or is it the effect of semaglutide without the use of rescue medication? Or is it another effect? Similarly, participants could discontinue randomised treatment. Is the true effect of treatment the effect of the decision to initiate treatment with semaglutide or is it the effect of semaglutide had all patients completed their prescribed course of treatment? Unless specified, we cannot understand and interpret the estimated treatment effect.

### ICH E9 (R1) definition of estimands

Pharmaceutical industry trials are governed by scientific guidelines produced by ICH (International Council for Harmonisation of technical requirements for pharmaceuticals for human use). Difficulties in expressing clearly the treatment effect to be estimated in clinical trials led to a new addendum to the ICH E9 guideline on statistical principles for clinical trials [3].

The ICH definition of an estimand requires a clear overview of intercurrent events and the associated strategy (see Table 1) chosen to reflect the clinical question of interest for each event. In addition, the estimand includes a complete description of the following attributes:

- *Treatment condition*, and as appropriate, the alternative treatment condition
- *Population* of patients targeted (e.g. patients with type 2 diabetes)
- *Variable* (or endpoint) obtained for each patient to address the clinical question (e.g. change from baseline to week 26 in HbA1C)
- *Population-level summary* for the variable, providing a basis for comparison between treatment conditions (e.g. difference in means in change from baseline to week 26 in HbA1C)

**Table 1:** Potential strategies for intercurrent events

**Strategy**	Relevant values for patients with the intercurrent event
Treatment policy	Actual values of the variable regardless of whether the intercurrent event has occurred
Composite	Modified definition of the variable incorporating the intercurrent event
Hypothetical	Values the variable would have taken in the hypothetical scenario envisaged in which the intercurrent event would not occur
While on-treatment/at-risk	Values of the variable up to the time of the intercurrent event
Principal stratum	Restrict population to patients who would not experience the intercurrent event

Strategies for intercurrent events can be incorporated in the treatment, population or variable attributes or can be specified separately. For example, “use of rescue medication as required” could be included as part of the treatment condition.

The PICO (population, intervention, control, and outcomes) format has often been used for framing clinical research questions [6]. Compared to PICO, the estimands framework in addition addresses the key issues of intercurrent events and summary measure, and without these elements, the treatment effect is not adequately defined.

In the PIONEER-1 example, employing a treatment policy strategy for rescue medication includes all data after the intercurrent event and estimates a treatment effect regardless of use of rescue medication, i.e. the treatment effect includes the impact of rescue medication on glycaemic control. A hypothetical strategy estimates the effect of semaglutide in the absence of rescue medication [7].

A composite strategy can be appropriate where the intercurrent event represents a poor (or positive) outcome of treatment. For example, the SYNAPSE trial compared mepolizumab and placebo in chronic rhinosinusitis with nasal polyps [8]. The co-primary endpoints were nasal polyps score and nasal blockage score at the end of the trial. A key intercurrent event was surgery for nasal polyps, a bad outcome for the patient that would be expected to subsequently improve nasal scores. A treatment policy strategy would include nasal scores after surgery in the comparison, but this would not reflect the negative outcome of surgery that was undertaken after treatment initiation. Instead, a composite strategy was used incorporating surgery as an adverse outcome in the endpoint definition.

### ITT and per-protocol analysis

Historically analyses have been viewed as a dichotomy: they are either ITT analyses or “per-protocol” [9]. For example, the current Cochrane Risk of Bias tool [10] defines in Sect. 1.3 only two possible treatment effects of interest:

1. The effect of assignment to the interventions at baseline (regardless of whether the interventions are received during follow-up, sometimes known as the ‘intention-to-treat effect’); or.
2. The effect of adhering to intervention as specified in the trial protocol (sometimes known as the ‘per-protocol effect’).

When defined as above, the intention-to-treat effect corresponds to a treatment policy strategy for all intercurrent events. ITT analysis is often interpreted as referring only to including all randomised patients in the population analysed but actually requires complete follow-up of all randomised patients to the end of the planned study period [11]. When using this strategy, it is important to ensure data are still collected after the intercurrent event as these data will be included in the analysis.

It is increasingly recognised that the treatment effect estimated by the treatment policy approach may not always be of primary clinical interest and may not appropriately communicate to prescribers and patients the efficacy that is directly attributable to the treatment, i.e. what can be expected in terms of efficacy if the patient takes the medication as prescribed [12–14].

The distinguishing feature of a per-protocol analysis is that patients with major protocol violations are excluded altogether and all of their data including data collected prior to the violation is not used. The decision on whether to exclude a patient based on their adherence to the protocol can be somewhat arbitrary. When a per-protocol analysis excludes patients who discontinue due to lack of efficacy, this information on poor efficacy is lost from the analysis. The clinical question that is being addressed by a per-protocol analysis is somewhat unclear [3]. By requiring that intercurrent events are defined along with their associated strategy, the estimand framework allows the corresponding clinical questions to be precisely defined. The terms ITT and per-protocol do not accurately describe the estimand.

The PIONEER-1 trial reported an estimand for the glycated haemoglobin endpoint that used a hypothetical strategy for discontinuation of trial medication. The analysis used all data until trial medication was discontinued and predicted data that would have been observed if treatment had not been discontinued. All patients were included in the analysis, so the estimated benefit is not the effect in the subset of patients who took the medication as intended, a known bias of previous per-protocol estimates of treatment effects [15].

The estimand framework distinguishes between the target of estimation (trial estimand) and the method of estimation (estimator). In some cases, the estimand is difficult to estimate reliably or a more sophisticated analysis is required. An explicit definition of the estimand allows a more transparent assessment of whether the estimation method appropriately addresses the clinical question of interest.

### Intercurrent events and missing data

There is an important distinction between an intercurrent event and missing data. Whether data are considered to be missing can depend on the choice of strategy for intercurrent events. For example, in the PIONEER-1 study a key intercurrent event was use of rescue medication. If data are unavailable for a particular participant following use of rescue medication, this data would be missing for a treatment policy strategy but not relevant for a hypothetical strategy (see Table 1). In contrast, if data are available, these data are relevant for a treatment policy strategy, but they are not relevant for a hypothetical strategy. For the hypothetical strategy, the relevant data will need to be predicted under the hypothetical scenario envisaged, typically by multiple imputation.

## Conclusions

The estimand framework provides an important approach to defining the treatment effect to be estimated in a clinical trial. Different treatment effects can be considered depending on how intercurrent events are included in the estimand definition and therefore there is no single “true” treatment effect.

Trials should be designed with a clearly articulated clinical question of interest. It is necessary to address intercurrent events when describing the clinical question of interest in order to precisely define the treatment effect that is to be estimated. Often, there will be multiple questions of interest, and these will lead to different estimands which result in different estimates of benefit.

A description of estimands should be included in trial publications to provide clarity on the treatment effect reported. An overview of the frequency and timing of each type of intercurrent event by treatment group is needed for proper interpretation of the estimated treatment effect. The estimand used by a trial is an important feature for meta-analyses as currently such analyses may combine estimates from different estimands when reporting treatment effects. We encourage revisions to methods recommended by the Cochrane group and to the CONSORT statement to reflect the centrality of estimands to clinical trials.

## Data Availability

Data sharing is not applicable to this article as no datasets were generated or analysed during the current study.

## Abbreviations

ICH: International Council for Harmonisation of technical requirements for pharmaceuticals for human use
PICO: Population, intervention, control, and outcomes
